# An In Vivo Method to Quantify Lymphangiogenesis in Zebrafish

**DOI:** 10.1371/journal.pone.0045240

**Published:** 2012-09-13

**Authors:** Scott J. Hoffman, Peter J. Psaltis, Karl J. Clark, Daniel B. Spoon, Colin D. Chue, Stephen C. Ekker, Robert D. Simari

**Affiliations:** 1 Division of Cardiovascular Diseases, Mayo Clinic, Rochester, Minnesota, United States of America; 2 Division of Biochemistry and Molecular Biology, Mayo Clinic, Rochester, Minnesota, United States of America; Texas A&M University, United States of America

## Abstract

**Background:**

Lymphangiogenesis is a highly regulated process involved in the pathogenesis of disease. Current in vivo models to assess lymphangiogenesis are largely unphysiologic. The zebrafish is a powerful model system for studying development, due to its rapid growth and transparency during early stages of life. Identification of a network of trunk lymphatic capillaries in zebrafish provides an opportunity to quantify lymphatic growth in vivo.

**Methods and Results:**

Late-phase microangiography was used to detect trunk lymphatic capillaries in zebrafish 2- and 3-days post-fertilization. Using this approach, real-time changes in lymphatic capillary development were measured in response to modulators of lymphangiogenesis. Recombinant human vascular endothelial growth factor (VEGF)-C added directly to the zebrafish aqueous environment as well as human endothelial and mouse melanoma cell transplantation resulted in increased lymphatic capillary growth, while morpholino-based knockdown of *vegfc* and chemical inhibitors of lymphangiogenesis added to the aqueous environment resulted in decreased lymphatic capillary growth.

**Conclusion:**

Lymphatic capillaries in embryonic and larval zebrafish can be quantified using late-phase microangiography. Human activators and small molecule inhibitors of lymphangiogenesis, as well as transplanted human endothelial and mouse melanoma cells, alter lymphatic capillary development in zebrafish. The ability to rapidly quantify changes in lymphatic growth under physiologic conditions will allow for broad screening of lymphangiogenesis modulators, as well as help define cellular roles and elucidate pathways of lymphatic development.

## Introduction

Lymphangiogenesis, the formation of new lymphatic vessels, is a highly regulated process and an important therapeutic drug target due its involvement in the pathogenesis of disease processes including inflammation, obesity, atherosclerosis, lymphedema, and cancer [1]–[4]. Studies to elucidate the underlying mechanisms of lymphatic development have identified vascular endothelial growth factor-C (VEGF-C) as a key activator of lymphangiogenesis that functions via VEGF receptor-3 (VEGFR-3) on the surface of lymphatic endothelial cells (LECs) [1], [2], [4]. However, a thorough understanding of pathways that regulate lymphangiogenesis has been limited, in part due to the lack of a rapid and physiologic in vivo method to precisely measure lymphatic development.

Traditional wound healing models of pathologic lymphangiogenesis rely on artificially-induced inflammation to stimulate lymphatic growth and often lead to potential confounding bystander effects from nearby pro-angiogenic or lymphangiogenic inflammatory cells [5]. Moreover, this approach involves immunostaining of LECs for lymphangiogenesis quantification and thus does not capture real-time changes in lymphatic growth. Techniques attempting to reduce bystander effects, as well as methods that employ in vivo imaging of lymphatic vessels, are often lengthy, complicated, expensive, and permit only small numbers of animals to be tested, thus limiting their use and accessibility [6]–[8].

In recent years, the zebrafish (*Danio rerio*) has emerged as a popular model to study vascular development. Zebrafish offer many advantages, including rapid extrauterine growth, prolific reproduction, and transparency during early stages of life allowing for real-time in vivo imaging of development. Additionally, due to conserved evolutionary pathways, numerous compounds specifically designed to be active in mammals cross-react with zebrafish, occasionally by merely being added to the zebrafish aqueous environment [9], [10].

To date, lymphangiogenesis studies in zebrafish have focused primarily on development of the thoracic duct [10], [11]. However, using lymphangiography, several additional lymphatic vessels in the zebrafish trunk have been identified including a complex network of blind-ended lymphatic capillaries by 18 days post-fertilization (dpf) [11]. The appearance of this capillary network is analogous to cells in tube formation assays used for in vitro quantification of lymphangiogenesis [12]; thus, we postulated that a similar experimental approach, involving morphometric analysis of in vivo lymphatic growth over time, could be applied to lymphatic capillaries in early zebrafish development when modifications to lymphatic growth may be most readily apparent. In this study, the latter was accomplished by using a microangiographic strategy to detect lymphatic capillaries in embryonic and early larval zebrafish exposed to various modulators of lymphangiogenesis.

## Methods

An expanded Methods section with associated references is available online (File S1).

### Ethics Statement

All animal experiments complied with the standards stated in the Guide for the Care and Use of Laboratory Animals (Institute of Laboratory Animal Resources, National Academy of Sciences, Bethesda, MD) and were approved by the Mayo Clinic Institutional Animal Care and Use Committee.

### Animals

Zebrafish experiments utilized the *Danio rerio* transgenic line *Tg(fli1:EGFP)^y1^*, expressing green fluorescent protein (GFP) in vascular endothelium, described previously [13]. Zebrafish were kept at 29°C in between experiments, unless otherwise indicated.

### Cells and Reagents

For experiments quantifying lymphatic capillary development, the following compounds were added directly to embryo water (15 mM NaCl, 0.5 mM KCl, 1.0 mM MgSO_4_, 0.15 KH_2_PO_4_, 0.05 mM Na_2_HPO_4_, 1.0 mM CaCl_2_, 0.7 mM NaHCO_3_) containing approximately 10–20 zebrafish: recombinant human VEGF-C (rhVEGF-C, R&D Systems, Minneapolis, MN), at a final concentration of 200 pg/ml (enhanced capillary growth) or 100 pg/ml (rescue of *vegfc* morpholino); rapamycin (Sigma-Aldrich, St. Louis, MO) in dimethyl sulfoxide (DMSO) at a final concentration of 400 nM; human VEGF receptor-3 (hVEGFR-3) kinase inhibitor (MAZ51; EMD Biosciences, San Diego, CA) in DMSO, at a final concentration of 30 µM. Equivalent volumes of DMSO were added to the embryo water of control zebrafish, corresponding to the volumes of rapamycin (1 µl) and hVEGFR-3 inhibitor (4 µl) used. All compounds were initially given on the day of fertilization (day 0) and subsequently replaced every 24 hours in fresh embryo water.

Lymphangiogenesis rescue experiments were conducted using three cultured cell lines: human umbilical vein endothelial cells (HUVECs; ATCC, Manassas, VA), B16 mouse melanoma cells (ATCC) and human embryonic kidney 293 cells (ATCC) [14]–[16]. HUVECs were cultured in endothelial growth medium-2 (Lonza, Walkersville, MD) supplemented with 2% fetal bovine serum (FBS). B16 and 293 cells were cultured in Dulbecco's Modified Eagle Medium (Mediatech Inc, Manassas, VA) supplemented with 10% FBS.

### Morpholino Injection

A morpholino antisense oligonucleotide used to inhibit translation of *vegfc* (5′-TGAGCAGAGTCTCTTGAAAGTTCCC-3′) was a gift from Dr. Stephen Ekker. Solutions were prepared and injected into zebrafish embryos on the day of fertilization (day 0) up to the 4-cell stage, as described [17]. The morpholino dose injected was 5 ng per embryo.

### Xenotransplantation

Tricaine MS-222 (0.04 mg/mL, Sigma-Aldrich)-anesthetized 2-dpf *Tg(fli1:EGFP)^y1^* zebrafish, previously-injected with a *vegfc* morpholino, were injected with 100–500 HUVECs, B16 or 293 cells labeled with CellTracker Orange (Invitrogen, Eugene, OR). Prior to injection, cells were washed and resuspended in 0.9x PBS supplemented with 0.3 U/µl heparin (APP Pharmaceuticals, LLC, Schaumburg, IL) and 0.1 U/µl DNase (Roche Diagnostics, Indianapolis, IN) to a final density of 2×10^5^/µl. Injections were performed using a PL1-90 microinjector (Harvard Apparatus, Holliston, MA) and borosilicate glass needles (1.5 mm outside diameter, no filament; World Precision Instruments, Sarasota, FL) made with a Flaming/Brown micropipette puller (Sutter Instruments, Novato, CA), as previously-described [18]. Transplanted zebrafish were kept overnight in embryo water at 37°C prior to confocal imaging the next day (3 dpf).

### Microangiography and Lymphangiography

Microangiography was performed as previously described [19]; however, 70 kilodalton (kDa) Texas Red-linked low molecular weight dextran (Texas Red-LMD, Invitrogen) was used for these studies. For early-phase microangiography, anesthetized zebrafish were imaged by confocal microscopy at 15 minutes post-injection to observe Texas Red-LMD within blood vessels. For late-phase microangiography, zebrafish were imaged at 4 hours post-injection, at which time Texas Red-LMD was visible within lymphatic vessels. For whole zebrafish imaging, 2000 kDa Fluorescein-linked high molecular weight dextran (Fluorescein-HMD; Invitrogen) was co-injected with Texas Red-LMD to enhance green fluorescence emitted by the GFP-expressing zebrafish and to expose significant vascular leaks.

Traditional lymphangiography was performed as described [11]. Imaging for early-phase and late-phase lymphangiography was performed at 15 minutes and 4 hours post-injection, respectively.

### In Vivo Microscopy

At 2 dpf or 3 dpf, Tricaine-anesthetized *Tg(fli1:EGFP)^y1^* zebrafish were imaged in embryo water using a Zeiss LSM780 inverted confocal microscope, equipped with argon and ultraviolet lasers for multicolor analyses. Images were focused on the deep lymphatic capillaries within the mid-trunk region.

Traditional two-channel modes were used to detect green and red fluorescence in EGFP-expressing zebrafish injected with Texas Red-LMD (488 nm filter for EGFP excitation, 561 nm filter for Texas-Red excitation). The spectral mode of Zeiss LSM780 was used to detect three fluorophores in EGFP-expressing zebrafish injected with both Texas Red-LMD and CellTracker Orange-labeled cells (594 nm filter for excitation), as previously described [20], [21]. CellTracker Orange-labeled cells were pseudo-colored blue to distinguish them from vessels containing Texas Red-LMD.

### Quantitative Method to Assess Lymphangiogenesis

For each *Tg(fli1:EGFP)^y1^* zebrafish analyzed, confocal images of the mid-trunk region spanning approximately 5–7 somitic interspaces (defined as the region between two consecutive somitic boundary clefts filled with Texas Red-LMD) were acquired for quantification. The area contained within four consecutive somitic interspaces (starting with the most anterior interspace) was used for lymphatic capillary quantification. Morphometric analyses were performed using MetaMorph microscopy automation and image analysis software (Molecular Devices, LLC, Sunnyvale, CA). The average total capillary length for each treatment group imaged over three or more independent experiments was subsequently calculated. The results were expressed as the mean lymphatic capillary length (µM) per unit area.

### Immunoblotting

Cell lysates were obtained from HUVECs, B16, and 293 cells using cell lysis buffer in the presence of protease inhibitors (Roche). Conditioned media was also collected. After centrifugation to remove cell debris, samples were denatured and then subjected to sodium dodecyl sulphate polyacrylamide gel electrophoresis under reducing conditions, followed by transfer onto a polyvinylidene fluoride membrane (Biorad Laboratories, Hercules, CA). After blocking with Tris-buffered saline-0.05%Tween containing 5% milk, blots were incubated with 1∶100 anti-VEGF-C antibody (sc-1881; Santa Cruz Biotechnology, Santa Cruz, CA) or 1∶2000 anti-α-Tubulin (T6199; Sigma-Aldrich), followed by incubation with appropriate secondary antibodies (Santa Cruz) and ECL chemiluminescence detection system (Fisher Pierce, Rockford, IL) as per the manufacturer's instructions.

### Bright-field and Fluorescence Microscopy

Bright-field, green, and red fluorescence images (5x) of untreated Tricaine-anesthetized *Tg(fli1:EGFP)^y1^* zebrafish as well as those injected with a *vegfc* morpholino or exposed to hVEGFR-3 inhibitor or rapamycin were taken in embryo water using a Zeiss Axio Observer.A1 inverted fluorescent microscope equipped with a Sony HDR-HC9 high-definition video camera.

### Statistical Analysis

Statistics were performed using GraphPad Prism 4 software. Results are expressed as mean ± SEM. Differences between groups were analyzed for statistical significance using an unpaired two-tailed Student *t* test.

## Results

### Detection of Deep Trunk Lymphatic Capillaries Early in Zebrafish Development

**Figure 1:**
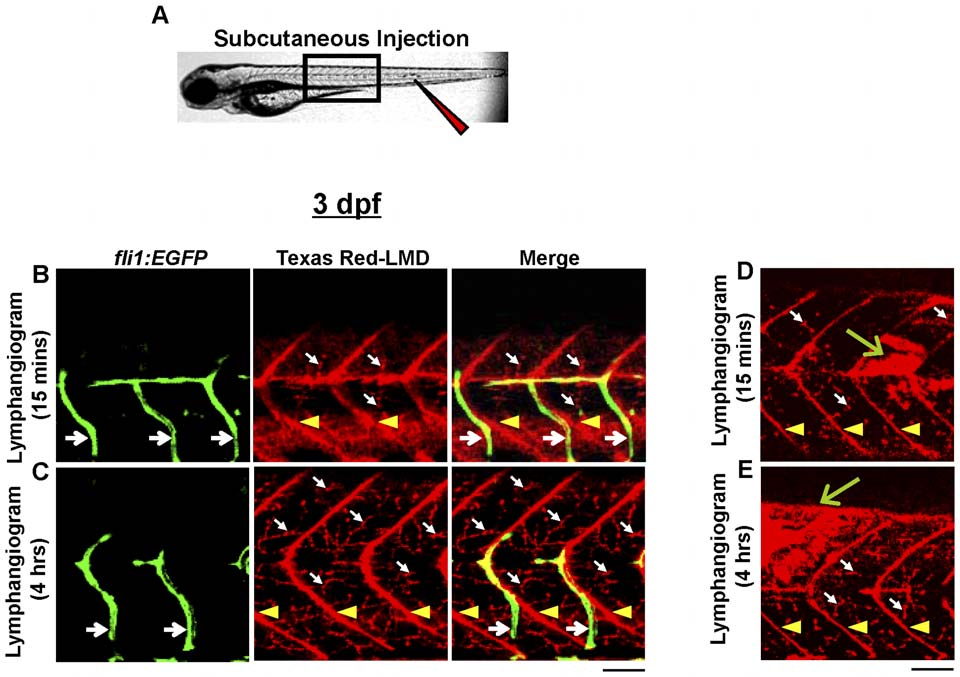
Lymphangiography reveals lymphatic capillaries in early zebrafish development. **A**, Subcutaneous injection technique employed for lymphangiography. The narrow red triangle represents the needle used to inject Texas Red-LMD into the zebrafish posterior tail. The black box demarcates the approximate region of the trunk imaged. **B** and **C**, Ideal lymphangiogram of 3-dpf *Tg(fli1:EGFP)^y1^* zebrafish (green blood vessels). **B**, At 15 minutes post-injection (early-phase), green ISVs (large white arrows) are distinguished from extravascular collections of Texas Red-LMD at somitic boundaries (yellow arrowheads) and small red lymphatic vessels (small white arrows) deep to and within the somitic interspaces. **C**, At 4 hours post-injection (late-phase), Texas Red-LMD is still not present within ISVs (large white arrows); however, more of the small lymphatics contain Texas Red-LMD and comprise an intricate array of capillaries (small white arrows). **D** and **E**, Typical lymphangiogram at 15 minutes and 4 hours post-injection, in which images are partially-obscured by blotches of Texas Red-LMD (green arrows) that diffused from the original subcutaneous site. Scale bars, 50 µm.

The presence of lymphatic capillaries in 3-dpf (larval) *Tg(fli1:EGFP)^y1^* zebrafish were confirmed by employing a lymphangiography technique previously used to identify trunk lymphatics in older zebrafish [11], in which fluorescently-linked dextran was injected subcutaneously into the posterior tail (Figure 1A). However, in this study, Texas Red-LMD was utilized rather than fluorescently-linked HMD as it was presumed that nascent lymphatic vessels at 3 dpf would more readily absorb LMD. Fifteen minutes post-injection (early-phase), confocal imaging of the mid-trunk region revealed chevron-shaped extravascular collections of Texas-Red LMD in somite boundary clefts [22], distinguished from intersegmental blood vessels (ISVs) expressing GFP in vascular endothelium (Figure 1B). Additionally, several deep lymphatic vessels were visible and appeared to be partially filled with Texas Red-LMD. By 4 hours post-injection (late-phase), significantly more of the deep lymphatics, but no ISVs, contained Texas Red-LMD and were seen to comprise an intricate array of blind-ended capillaries (Figure 1C), corresponding to the lymphatic network previously observed in 18-dpf zebrafish [11].

Unexpectedly, these initial experiments revealed a potential drawback of lymphangiography as a technique for lymphatic capillary visualization; namely, the frequent appearance of excess Texas Red-LMD diffusing from subcutaneous injection sites as blotches that obscure lymphatic capillaries (Figure 1D, E). Thus, an alternative imaging method that would unambiguously reveal lymphatic capillaries for quantification was sought. It was discovered that late-phase microangiography with Texas Red-LMD was one such approach.

**Figure 2:**
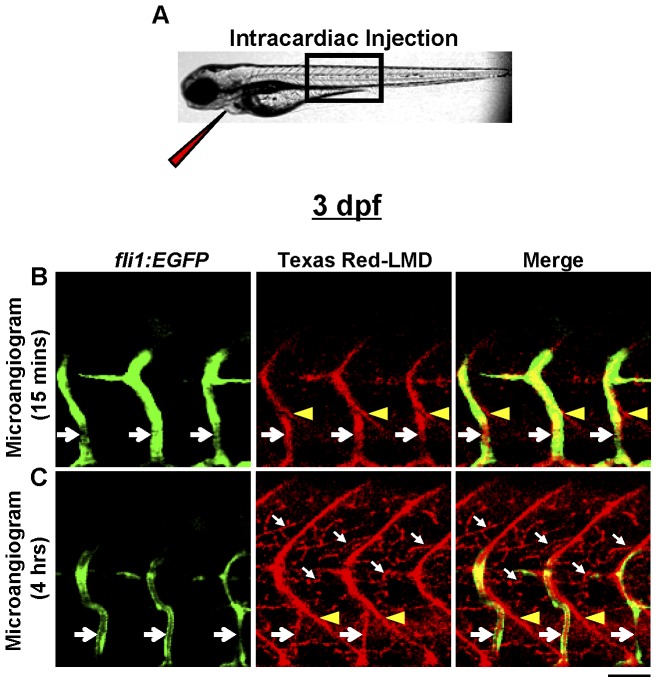
Late-phase microangiography clearly demonstrates lymphatic capillaries in early zebrafish development. **A**, Intracardiac injection technique employed for microangiography. The narrow red triangle represents the needle used to inject Texas Red-LMD into the zebrafish heart. The black box demarcates the approximate region of the trunk imaged. **B** and **C**, Microangiogram of 3-dpf *Tg(fli1:EGFP)^y1^* zebrafish. **B**, At 15 minutes post-injection (early-phase), Texas Red-LMD is primarily within ISVs (large white arrows), and lymphatic capillaries contain no Texas Red-LMD. **C**, By 4 hours post-injection (late-phase), most Texas Red-LMD has leaked from the blood vessels and collected at somitic boundaries (yellow arrowheads) or within deep lymphatic capillaries (small white arrows). Scale bar, 50 µm.

Microangiography was performed by intracardiac injection of Texas-Red LMD into 3-dpf *Tg(fli1:EGFP)^y1^* zebrafish (Figure 2A). Fifteen minutes post-injection (early-phase), Texas Red-LMD was contained almost exclusively within ISVs and not in any putative lymphatic capillaries (Figure 2B), further specifying the lymphatic origin of the latter. However, due to the small size of Texas Red-LMD and considerable permeability of developing blood vessels in 3-dpf zebrafish, most Texas Red-LMD had leaked from the GFP-expressing vasculature by 4 hours post-injection (late-phase) and either collected at somite boundaries or was absorbed by lymphatic capillaries, essentially simulating a late-phase lymphangiogram (Figure 2C). Thus, late-phase microangiography, a technique that requires approximately the same amount of time as lymphangiography to clearly visualize lymphatic capillaries but does so more reliably with the added advantage of illuminating blood vessels, was employed for all subsequent experiments.

### Zebrafish Lymphatic Capillary Development is Regulated by VEGF-C

**Figure 3:**
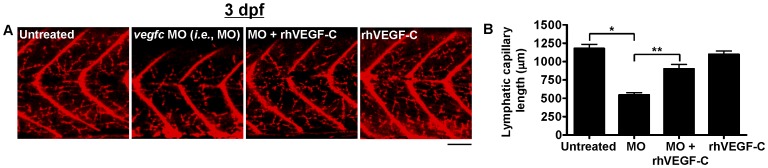
Quantification of lymphatic capillary development after knockdown with a *vegfc* morpholino. Red fluorescent images from late-phase microangiograms of *Tg(fli1:EGFP)^y1^* zebrafish are shown, highlighting Texas Red-LMD uptake within lymphatic capillaries. Results of quantitative morphometric analyses are displayed in bar graphs. **A** and **B**, Zebrafish injected with a *vegfc* morpholino (MO) have significant inhibition of lymphatic capillary growth at 3 dpf (n = 32) compared to untreated zebrafish (n = 32) or those exposed to rhVEGF-C (n = 14), which is mitigated by the addition of rhVEGF-C (100 pg/ml) to the aqueous environment (n = 15). *P<0.0001, **P<0.0001. Scale bars, 50 µm.

To determine if zebrafish lymphatic capillary growth is mediated by known activators of lymphangiogenesis, zebrafish embryos were injected with a morpholino, previously shown to specifically inhibit the *vegfc* ortholog in zebrafish [10], [11]. Knockdown of *vegfc* expression induced a significant decrease in lymphatic capillary growth compared to untreated zebrafish at 3 dpf (Figure 3A, B), while only causing other mild phenotypic changes such as trace edema and occasional small blood vessel defects (Figure S1), indicating that *vegfc* is critical for lymphatic capillary development. Specificity of *vegfc* knockdown was confirmed by a rescue experiment in which exogenous rhVEGF-C (a prospective treatment for human lymphedema [23]) restored lymphatic capillary growth by 3 dpf when added to the aqueous environment of *vegfc* morphant zebrafish (Figure 3A, B).

**Figure 4:**
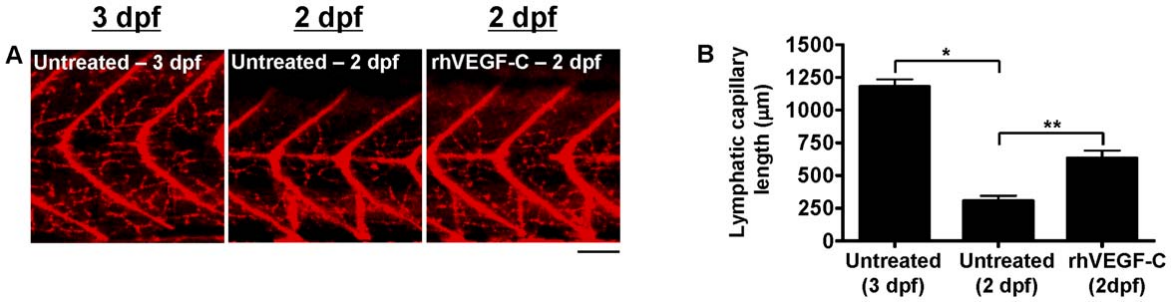
Quantification of lymphatic capillary development after stimulation with rhVEGF-C. Red fluorescent images from late-phase microangiograms of *Tg(fli1:EGFP)^y1^* zebrafish are shown, highlighting Texas Red-LMD uptake within lymphatic capillaries. Results of quantitative morphometric analyses are displayed in bar graphs. **A** and **B**, At 2 dpf, fewer lymphatic capillaries are present in untreated zebrafish (n = 18), compared to 3-dpf untreated zebrafish (n = 32), but they increase significantly with the addition of rhVEGF-C to the zebrafish aqueous environment (n = 18). *P<0.0001, **P<0.0001. Scale bars, 50 µm.

The effect of rhVEGF-C on lymphatic capillary development in non-morphant zebrafish was also assessed. Similar to a previous study examining the effect of rhVEGF on vascular development in embryonic zebrafish [9], rhVEGF-C was added daily to the zebrafish aqueous environment, starting at approximately 1 hour post-fertilization (hpf). Lymphatic capillary growth was subsequently quantified by morphometric analyses of late-phase microangiograms at 2-dpf (late embryonic stage), since, at baseline, 2-dpf zebrafish demonstrate substantially fewer lymphatic capillaries than 3-dpf zebrafish (Figure 4A, B). Consequently, 2-dpf zebrafish exposed to rhVEGF-C exhibited a significant increase in lymphatic capillary growth compared to untreated 2-dpf zebrafish (Figure 4A, B), but no detectable increase in lymphatic capillary growth above baseline at 3-dpf (Figure 3A). The lack of sustained activation of lymphangiogenesis by rhVEGF-C is uncertain but could be related to decreased permeability of the zebrafish integument by the time of hatching (approximately 50 hpf), inhibiting further rhVEGF-C absorption, or perhaps an intrinsic zebrafish homeostatic mechanism, preventing potentially harmful excessive lymphatic growth at 3 dpf.

### Suppression of Zebrafish Lymphatic Capillary Development by Small Molecules

**Figure 5:**
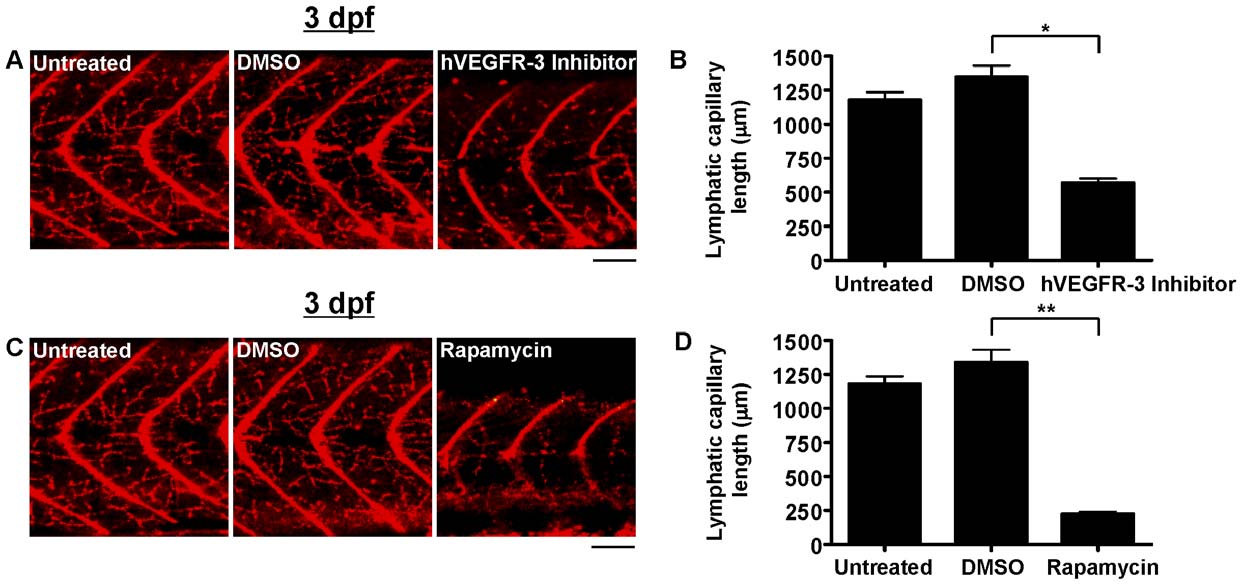
Quantification of lymphatic capillary development after inhibition with small molecules. Red fluorescent images from late-phase microangiograms of *Tg(fli1:EGFP)^y1^* zebrafish are shown, highlighting Texas Red-LMD uptake within lymphatic capillaries. Results of quantitative morphometric analyses are displayed in bar graphs. **A** and **B**, Addition of hVEGFR-3 inhibitor in DMSO (n = 30) or rapamycin in DMSO (n = 18) to the aqueous environment significantly reduced lymphatic capillary development at 3 dpf, compared to zebrafish exposed to DMSO alone (n = 21 and 15, respectively). *P<0.0001, **P<0.0001. Scale bars, 50 µm.

Zebrafish lymphatic capillary development in the presence of chemical inhibitors of lymphatic growth was assessed. The hVEGFR-3 inhibitor (MAZ51) was added to the zebrafish aqueous environment at a dose (30 µM) reported to specifically inhibit lymphangiogenesis in human cells in vitro and Xenopus tadpoles in vivo [24], [25] and resulted in significantly fewer lymphatic capillaries than untreated zebrafish by 3 dpf (Figure 5A, B) but virtually no other detectable phenotypic changes (Figure S1). Zebrafish exposed to rapamycin, an in vivo inhibitor of lymphangiogenesis in both humans and zebrafish [10], [26]–[30], at a dose (400 nM) reported to suppress zebrafish thoracic duct development [10], exhibited a particularly striking inhibition of lymphatic capillary growth by 3 dpf (Figure 5C, D). It is unclear if this inhibition was related to a direct or indirect effect on lymphatic capillary development, as zebrafish exposed to rapamycin were markedly smaller than untreated zebrafish at 3 dpf; nonetheless, the other phenotypic features of rapamycin-treated zebrafish suggested successful targeting of lymphangiogenesis pathways, including large pericardial and yolk sac edema as well as increased vascular permeability (Figure S1), which were not improved by the addition of rhVEGF-C (data not shown).

### Zebrafish Lymphatic Capillary Development is Stimulated by Human Endothelial and Mouse Melanoma Cells

**Figure 6:**
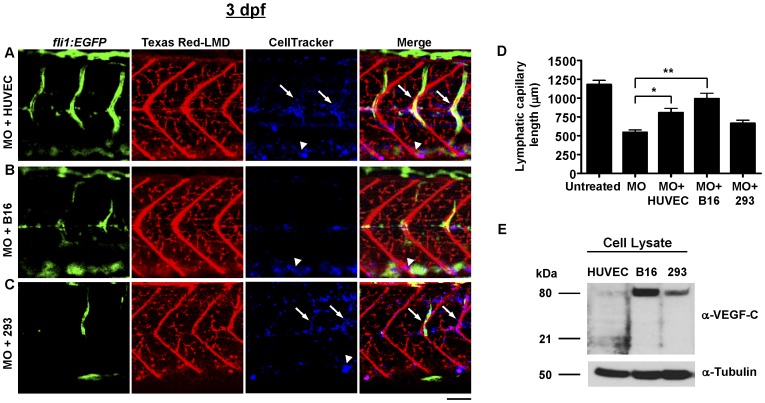
Quantification of lymphatic capillary development after xenotransplantation. Green fluorescent images (*fli1:EGFP*) reveal blood vessels, red fluorescent images (Texas Red-LMD) reveal lymphatic capillaries, and blue fluorescent images (CellTracker) reveal transplanted cells in 3-dpf *Tg(fli1:EGFP)^y1^* zebrafish. Results of quantitative morphometric analyses are displayed in bar graphs. **A–D**, At 3 dpf (n = 32), the inhibitory effect of the *vegfc* morpholino MO (n = 32) was rescued by transplantation of HUVECs (n = 18) and B16 cells (n = 20), but not 293 cells (n = 25). *P = 0.0005, **P<0.0001. Scale bars, 50 µM. All cell types (blue) were detected in major blood vessels, including the dorsal aorta (white arrowheads). HUVECs and 293 cells were also seen extravascularly, bordering lymphatic capillaries (white arrows). Note: fewer ISVs (green) in images of B16- or 293-injected morphants relates to the particular focal plane imaged, since alternative focal planes revealed approximately normal numbers of ISVs in these and other similarly-treated zebrafish (data not shown). **E**, Immunoblot to detect human VEGF-C in cultured HUVEC, B16, and 293 cells. The active, proteolytically-processed form of VEGF-C (21 kilodaltons, kDa) was only detected at appreciable levels in HUVEC lysate. B16 and 293 cell lysates contained VEGF-C in a dimerized precursor form (80 kDa). No VEGF-C was detected in conditioned media from any of the cells (data not shown). An antibody against α-tubulin was used as a loading control.

Lymphatic capillary development in zebrafish transplanted with human endothelial cells, known to secrete numerous VEGF growth factors [31], and murine cancer cells, which may facilitate metastasis by activating lymphatic growth [1], was assessed. Xenotransplantation is an important technique to study cell function in zebrafish, made possible by the fact that the latter do not have an effective immune response during early development and thus do not undergo transplant rejection [18]. The cells are typically given by intracardiac injection beyond 48 hpf to ensure adequate development of the cardiovascular system. Thus, to improve the likelihood of detecting an increase in lymphatic capillary growth, a *vegfc* morpholino was injected prior to transplantation. At 2-dpf, *vegfc* morphants were transplanted with human endothelial cells (HUVECs), as well as mouse melanoma cells (B16) and human embryonic kidney (293) cells as controls, via intracardiac injection. After overnight incubation of transplanted zebrafish at 37°C, all three injected cell types appeared to have increased in number relative to the original amount of cells injected and had taken up residence in the major blood vessels, but human endothelial and kidney cells also appeared extravascularly in the deep tissues bordering lymphatic capillaries (Figure 6A–C). Notably, morphant zebrafish transplanted with human endothelial and mouse melanoma cells, but not human kidney cells, had significantly more lymphatic capillary growth than untransplanted morphants (Figure 6D); however, the level of rescue did not correlate directly with the level of activated VEGF-C expression *in vitro* (Figure 6E).

## Discussion

Lymphangiogenesis is involved in the pathogenesis of numerous disease processes, including lymphedema, inflammation, obesity, atherosclerosis, and cancer. However, our current understanding of the mechanisms underlying lymphatic development is limited because of inadequate animal model systems to precisely measure lymphatic growth, impeding both the advancement of basic scientific research as well as the discovery of new drug targets. In the present study, this technical shortcoming is addressed through the development of an in vivo method to quantify lymphangiogenesis in zebrafish.

Previous lymphatic studies in zebrafish have concentrated primarily on development of the thoracic duct, which provides a qualitative measure of lymphangiogenesis but is not suitable to quantify changes in lymphatic growth [10], [11]. In this study, trunk lymphatic capillaries, resembling cells in tubulogenesis assays amenable to morphometric analyses, were assessed. Using fluorescently-linked LMD and late-phase microangiography, a complementary imaging approach to lymphangiography, zebrafish trunk lymphatic capillaries were detected in embryonic and early larval stages. This is the first report in which trunk lymphatic capillaries were visualized as early as 2 dpf. Prior investigations employing lymphangiography failed to detect lymphatic capillaries until several weeks post-fertilization, perhaps related to the use of fluorescently-linked HMD that may not be absorbed by developing lymphatic capillaries [11].

In this study, detection of early lymphatic capillary growth, a dynamic but predictable physiologic process, allowed for the quantification of lymphatic development in zebrafish. Human modulators of lymphangiogenesis, including an activator (rhVEGF-C) and multiple small molecule inhibitors (hVEGFR-3 inhibitor and rapamycin), reproducibly and specifically altered lymphatic capillary development, and human endothelial and mouse melanoma cells transplanted into zebrafish stimulated a measurable increase in lymphatic capillary growth after suppression with a *vegfc* morpholino. These findings underscore several key advantages of using the zebrafish model to measure lymphangiogenesis.

One advantage is the ability to detect real-time changes in lymphatic growth in large numbers of zebrafish after the addition of protein or chemical lymphangiogenesis modulators to the aqueous environment. The latter is the simplest route of delivery used in any in vivo model of lymphangiogenesis and leads to detectable increases or decreases in lymphatic growth within a few days. In contrast, many current methods for quantifying pathologic lymphangiogenesis permit only small numbers of animals to be tested, because they require complex surgical techniques such as suturing or pellet implantation of the mouse cornea in order to observe a change in lymphatic development that frequently does not occur for 1 or 2 weeks [5], [6], [32]. Moreover, these assays are useful only to study activation and not inhibition of lymphangiogenesis, since they rely on tissues that are initially devoid of lymphatics, and also provide instantaneous rather than real-time images of lymphatic growth, since immunostaining of selected tissue sections is required.

Another advantage of the zebrafish model is the ability to assess the effects of cells derived from multiple species on lymphangiogenesis in vivo, since zebrafish lack a functional immune system during embryonic and early larval stages. To our knowledge, this is the first report of xenotransplantation as a method to evaluate cellular roles in lymphatic development. Many cell types are believed to influence lymphangiogenesis, ranging from vascular endothelial cells primarily during embryonic development and inflammation, to cancer cells undergoing metastasis [1], [2]. Previously, human endothelial cells were presumed to have a stimulatory effect on lymphangiogenesis based solely on their ability to express activated VEGF-C and stimulate LECs in vitro [33], while mouse melanoma cells were considered to be only slightly lymphangiogenic based on a lack of activated VEGF-C production in vitro and minimal stimulation of lymphangiogenesis in vivo in murine studies of melanoma [34]. In this report, both cell types were capable of rescuing lymphatic growth in zebrafish following knockdown of *vegfc*, a particularly impressive finding given the fact that these morphants were less likely to thrive and develop under the extremely warm conditions used to maintain viability of transplanted cells. Thus, human endothelial cells, as well as mouse melanoma cells under the correct physiologic conditions, are capable of stimulating lymphatic development in vivo. Interestingly, mouse melanoma cells stimulated lymphatic growth to a greater degree than human endothelial cells in zebrafish, but the level of stimulation did not correlate directly with in vitro production of biologically-active VEGF-C, since only cultured human endothelial cells expressed the fully processed form of the protein. Mouse melanoma cells may therefore promote lymphangiogenesis through an alternative pathway to the VEGF-C/VEGFR-3 axis. Additional studies combining morpholino gene silencing with xenotransplantation may help elucidate the mechanisms by which human endothelial cells and mouse melanoma cells (as well as other endothelial and cancer cell types) stimulate lymphangiogenesis in zebrafish.

Despite many advantages, the method presented in this study does have distinct requirements. A moderate amount of technical expertise is required to perform injections in embryonic and larval zebrafish during microangiography, lymphangiography, and xenotransplantation experiments. Additionally, in vivo imaging is a rate-limiting step and requires the appropriate confocal microscopy equipment and software to visualize lymphatic capillaries early in development or perform 3-color spectral analyses after cell transplantation. The assay is temporally constrained by specific time-points at which injections, treatments, and imaging must occur and potentially confounded by variability in lymphatic capillary growth patterns occurring among different clutches of zebrafish embryos; although, the latter should be compensated by the ability to test large numbers of animals with this assay.

In summary, this is the first report of an in vivo method to quantify lymphangiogenesis in zebrafish. Contrary to most currently available models, the zebrafish model presented here is physiologic and permits large numbers of animals to be tested. Additionally, it is simple in design, provides rapid results, involves real-time imaging, and can be used to test the effects of numerous cell types on lymphatic growth. The ability to rapidly measure changes in lymphangiogenesis under physiologic conditions has broad implications. It opens the door to phenotypic characterization studies of lymphatic development in zebrafish, in conjunction with the large array of established mutant and transgenic lines, and will enable screening of additional lymphangiogenesis modulators. Moreover, it has the potential to help clarify mechanisms by which numerous cells alter lymphatic growth as well as elucidate pathways of lymphangiogenesis, the results of which will advance our understanding of disease.

## Supporting Information

Figure S1 **Zebrafish phenotype after** ***vegfc*** **morpholino injection or exposure to rapamycin or VEGFR-3 inhibitor.** **A–E**, Representative bright field and green or red fluorescence microscopy images of whole *Tg(fli1:EGFP)^y1^* zebrafish after microangiography with Texas Red-LMD and Fluorescein-HMD*, including untreated 2-dpf and 3-dpf zebrafish, or 3-dpf zebrafish subjected to (**C**) *vegfc* MO (5 ng), (**D**) hVEGFR-3 inhibitor (30 mM) or (**E**) rapamycin (400 ng/ml). Note, all lymphangiogenesis inhibitors (**C–E**) led to pericardial edema (black arrows), but it was most prominent in zebrafish exposed to rapamycin. The latter also had significant yolk sac edema (black arrowhead), and an apparent collection of fluorescently-linked dextran in the posterior trunk (white arrows), suggesting a blood vessel leak versus lymphedema. *Fluorescein-HMD was co-injected with Texas Red-LMD during microangiography to enhance the green fluorescent signal emitted by the GFP-expressing zebrafish, as well as expose significant vascular leaks.
(TIF)

File S1 **Detailed methods.**
(DOC)
