# Supplementary figures and images for: An In Vivo Method to Quantify Lymphangiogenesis in Zebrafish
Source: PLoS One. 2012 Sep 13;7(9):e45240. doi: 10.1371/journal.pone.0045240 (PMC3441694; doi:10.1371/journal.pone.0045240)

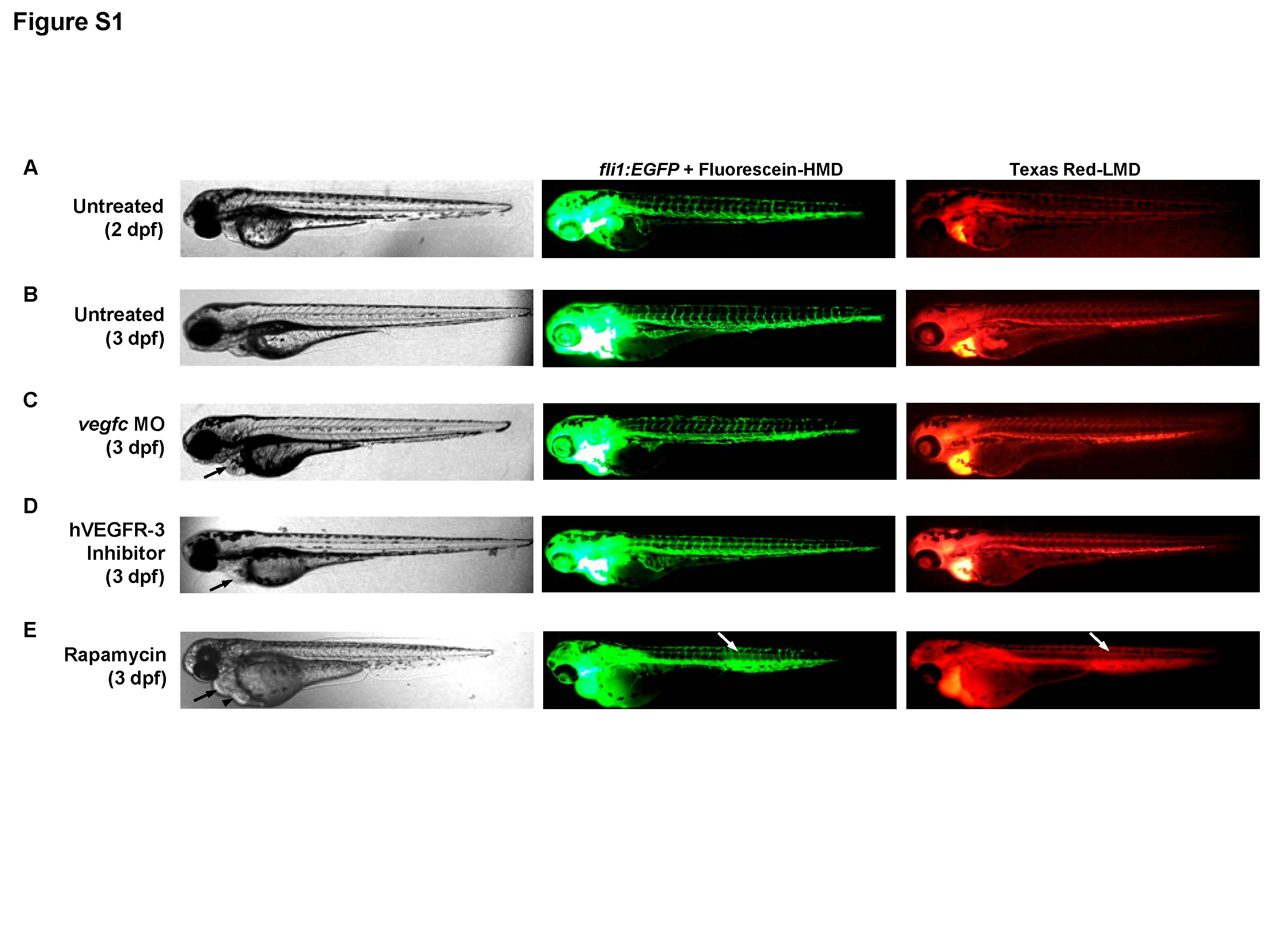

Supplement: Figure S1 — Zebrafish phenotype after vegfc morpholino injection or exposure to rapamycin or VEGFR-3 inhibitor. A–E, Representative bright field and green or red fluorescence microscopy images of whole Tg(fli1:EGFP)y1 zebrafish after microangiography with Texas Red-LMD and Fluorescein-HMD*, including untreated 2-dpf and 3-dpf zebrafish, or 3-dpf zebrafish subjected to (C) vegfc MO (5 ng), (D) hVEGFR-3 inhibitor (30 mM) or (E) rapamycin (400 ng/ml). Note, all lymphangiogenesis inhibitors (C–E) led to pericardial edema (black arrows), but it was most prominent in zebrafish exposed to rapamycin. The latter also had significant yolk sac edema (black arrowhead), and an apparent collection of fluorescently-linked dextran in the posterior trunk (white arrows), suggesting a blood vessel leak versus lymphedema. *Fluorescein-HMD was co-injected with Texas Red-LMD during microangiography to enhance the green fluorescent signal emitted by the GFP-expressing zebrafish, as well as expose significant vascular leaks. (TIF) [file pone.0045240.s001.tif]
