# Supplementary material for: An In Vivo Method to Quantify Lymphangiogenesis in Zebrafish
Source: PLoS One. 2012 Sep 13;7(9):e45240. doi: 10.1371/journal.pone.0045240 (PMC3441694; doi:10.1371/journal.pone.0045240)
Supplement: File S1 — Detailed methods. (DOC) [file pone.0045240.s002.doc]

**File S1.**

**METHODS**

**Xenotransplantation**

Tricaine MS-222 (0.04 mg/mL, Sigma-Aldrich)-anesthetized 2-dpf *Tg(fli1:EGFP)^y1^* zebrafish, previously-injected with a *vegfc* morpholino, were injected with 100–500 HUVECs, B16 or 293 cells labeled with CellTracker Orange (Invitrogen, Eugene, OR). After CellTracker Orange labeling, cells were washed and resuspended in 0.9x PBS supplemented with 0.3 U/µl heparin (APP Pharmaceuticals, LLC, Schaumburg, IL) and 0.1 U/µl DNase (Roche Diagnostics, Indianapolis, IN) to a final density of 2x10^5^/µl. Injections were performed using a PL1-90 microinjector (Harvard Apparatus, Holliston, MA) and borosilicate glass needles (1.5 mm outside diameter, no filament; World Precision Instruments, Sarasota, FL) made with a Flaming/Brown micropipette puller (Sutter Instruments, Novato, CA), as previously-described [1]. Five to 10 µl of each cell suspension was back-loaded into the needle and injected into the sinus venosus of the zebrafish heart by forced air. Xenotransplantation could be performed on up to 100 or more zebrafish per injection session (approximately 2 hours). Transplanted zebrafish were kept overnight in embryo water at 37ºC prior to confocal imaging the next day (3 dpf).

**Microangiography and Lymphangiography**

Microangiography was performed as previously described [2]; however, 70 kilodalton (kDa) Texas Red-linked low molecular weight dextran (Texas Red-LMD, Invitrogen) was used for these studies. Texas Red-LMD was solubilized in embryo water at 2 mg/ml concentration. Approximately 1.5 nl of the prepared solution was injected into the sinus venosi of 2- or 3-dpf Tricaine-anesthetized, transplanted or untransplanted *Tg(fli1:EGFP)^y1^* zebrafish, with or without prior morpholino injection. For early-phase microangiography, anesthetized zebrafish were imaged by confocal microscopy at 15 minutes post-injection, to observe Texas Red-LMD within blood vessels. For late-phase microangiography, zebrafish were imaged at 4 hours post-injection, at which time Texas Red-LMD was visible within lymphatic vessels. Of note, sub-optimal injection of Texas Red-LMD into the pericardial sac (no heart muscle penetration) frequently resulted in adequate late-phase images for lymphatic capillary quantification, perhaps through absorption of Texas Red-LMD by local lymphatics. For whole zebrafish imaging, 2000 kDa Fluorescein-linked high molecular weight dextran (Fluorescein-HMD; Invitrogen) was co-injected with Texas Red-LMD to enhance green fluorescence emitted by the GFP-expressing zebrafish and to expose significant vascular leaks (Figure S1). Generally, microangiography could be performed on 100 or more zebrafish per injection session (approximately 1–2 hours).

Traditional lymphangiography was performed as described [3]. Briefly, approximately 1 nl of Texas Red-LMD (2 mg/ml) was injected subcutaneously into the posterior tail of 2- or 3-dpf Tricaine-anesthetized zebrafish. Imaging for early-phase and late-phase lymphangiography was performed at 15 minutes and 4 hours post-injection, respectively.

**In Vivo Microscopy**

At 2 dpf or 3 dpf, Tricaine-anesthetized *Tg(fli1:EGFP)^y1^* zebrafish were imaged in embryo water using a Zeiss LSM780 inverted confocal microscope, equipped with argon and ultraviolet lasers for multicolor analyses. Images were focused on lymphatic capillaries within the mid-trunk region, spanning approximately 5–7 somitic interspaces (defined as the region between two consecutive somitic boundary clefts filled with Texas Red-LMD), from the posterior edge of the yolk sac to the second somite posterior to the tail end of the developing gut (Figure 1A).

Traditional two-channel modes were used to detect green and red fluorescence in EGFP-expressing zebrafish injected with Texas Red-LMD (488 nm filter for EGFP excitation, 561 nm filter for Texas-Red excitation). As described previously [4,5], the spectral mode of Zeiss LSM780 was used to detect three fluorophores in EGFP-expressing zebrafish injected with both Texas Red-LMD and CellTracker Orange-labeled cells (594 nm filter for excitation). Spectral images were obtained in a single scan with a 32-channel PMT detector coupled with built-in unmixing software. Spectral characteristics of single fluorophores (EGFP- endothelial cells, CellTracker Orange- injected cells, and Texas Red-LMD) in zebrafish were obtained and an unmixing algorithm was designed to separate the three colors in the same sample from a spectral image. CellTracker Orange-labeled cells were subsequently pseudo-colored blue, to distinguish them from vessels containing Texas Red-LMD. Using this method, approximately 70 zebrafish could be imaged per 3-hour session of microscopy (compared to approximately 10 corneal samples over 6–8 hours of imaging in a recent murine study, updating a traditional technique of pathologic lymphangiogenesis [6]).

**Quantitative Method to Assess Lymphangiogenesis**

For each *Tg(fli1:EGFP)^y1^* zebrafish analyzed, confocal images of the mid-trunk region, spanning approximately 5–7 somitic interspaces, were acquired for quantification. The area contained within four consecutive somitic interspaces (starting with the most anterior interspace) was used for lymphatic capillary quantification. Three blinded observers performed morphometric analyses by manually tracing each lymphatic capillary imaged within the four consecutive interspaces and computing the total length traced using MetaMorph microscopy automation and image analysis software (Molecular Devices, LLC, Sunnyvale, CA). The average total capillary length for each treatment group imaged over three or more independent experiments (n=15–32 total zebrafish per group) was subsequently calculated. The results were expressed as the mean lymphatic capillary length (µM) per unit area (the area contained within 4 consecutive somitic interspaces).

**REFERENCES**

1. Traver D, Paw BH, Poss KD, Penberthy WT, Lin A, et al. (2003) Transplantation and in vivo imaging of multilineage engraftment in zebrafish bloodless mutants. Nat Immunol 4: 1238-1246.

2. Weinstein BM, Stemple DL, Driever W, Fishman MC. (1995) *gridlock*, a localized heritable vascular patterning defect in the zebrafish. Nat Med 1: 1143-1147.

3. Yaniv K, Isogai S, Castranova D, Dye L, Hitomi J, Weinstein BM. (2006) Live imaging of lymphatic development in zebrafish. Nat Med 12: 711-716.

4. Pozzoli O, Vella P, Iaffaldano G, Parente V, Devanna P, et al. (2011) Endothelial fate and angiogenic properties of human CD34+ progenitor cells in zebrafish. Arterioscler Thromb Vasc Biol 31: 1589-1597.

5. Stoletov K, Fang L, Choi SH, Hartvigsen K, Hansen LF, et al. (2009) Vascular lipid accumulation, lipoprotein oxidation, and macrophage lipid uptake in hypercholesterolemic zebrafish. Circ Res 104: 952-960.

6. Cao R, Lim S, Ji H, Zhang Y, Yang Y, et al. (2011) Mouse corneal lymphangiogenesis model. Nat Protoc 6: 817-826.
